# 4′-Formyl­benzo-15-crown-5

**DOI:** 10.1107/S1600536808022186

**Published:** 2008-07-19

**Authors:** Conrad Fischer, Stefanie F. Helas, Wilhelm Seichter, Edwin Weber, Bakhtiyar T. Ibragimov

**Affiliations:** a Institut für Organische Chemie, TU Bergakademie Freiberg, Leipziger Strasse 29, D-09596 Freiberg/Sachsen, Germany; b Institute of Bioorganic Chemistry, Academy of Sciences of Uzbekistan, H Abdullaev 83, Tashkent 100125, Uzbekistan

## Abstract

In the title compound (systematic name: 17-formyl-2,5,8,11,14-penta­oxabicyclo­[13.4.0]nona­deca-15,17,19-triene), C_15_H_20_O_6_, the 15-crown-5 ring adopts a twisted conformation. The formyl group is coplanar with the benzene ring. The crystal packing is stabilized by C—H⋯O inter­actions involving the C=O group and ether O atoms as acceptors and methyl­ene CH groups as donors.

## Related literature

The unsubstituted benzocrown ether was characterized by Pedersen (1967 ▶) and its structure was described by Hanson (1978 ▶), while Rogers and co-workers reported 4′-amino- and 4′-nitro-substituted compounds (Rogers, Huggins *et al.*, 1992 ▶; Rogers, Henry & Rollins, 1992 ▶). For the synthesis of the title compound, see: Hyde *et al.* (1978 ▶).
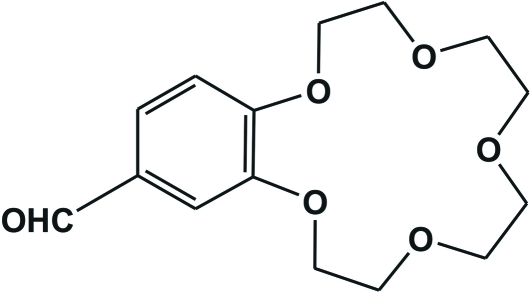


## Experimental

### 

#### Crystal data

C_15_H_20_O_6_
*M* *_r_* = 296.31
Monoclinic, 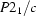

*a* = 18.0091 (8) Å
*b* = 9.6678 (4) Å
*c* = 8.1028 (3) Å
β = 91.262 (2)°
*V* = 1410.42 (10) Å^3^
*Z* = 4
Mo *K*α radiation
μ = 0.11 mm^−1^
*T* = 90 (2) K
0.60 × 0.39 × 0.05 mm

#### Data collection

Bruker Kappa APEXII CCD diffractometer
Absorption correction: multi-scan (*SADABS*; Sheldrick, 2004 ▶) *T*_min_ = 0.857, *T*_max_ = 0.995
18190 measured reflections
4523 independent reflections
3716 reflections with *I* > 2σ(*I*)
*R*_int_ = 0.026

#### Refinement

*R*[*F* ^2^ > 2σ(*F* ^2^)] = 0.037
*wR*(*F* ^2^) = 0.116
*S* = 1.00
4523 reflections
190 parameters
H-atom parameters constrained
Δρ_max_ = 0.28 e Å^−3^
Δρ_min_ = −0.22 e Å^−3^

### 

Data collection: *APEX2* (Bruker, 2004 ▶); cell refinement: *SAINT* (Bruker, 2004 ▶); data reduction: *SAINT*; program(s) used to solve structure: *SHELXS97* (Sheldrick, 2008 ▶); program(s) used to refine structure: *SHELXL97* (Sheldrick, 2008 ▶); molecular graphics: *ORTEP-3* (Farrugia, 1997 ▶); software used to prepare material for publication: *SHELXL97*.

## Supplementary Material

Crystal structure: contains datablocks global, I. DOI: 10.1107/S1600536808022186/gk2144sup1.cif

Structure factors: contains datablocks I. DOI: 10.1107/S1600536808022186/gk2144Isup2.hkl

Additional supplementary materials:  crystallographic information; 3D view; checkCIF report

## Figures and Tables

**Table 1:** Hydrogen-bond geometry (Å, °)

*D*—H⋯*A*	*D*—H	H⋯*A*	*D*⋯*A*	*D*—H⋯*A*
C13—H13*B*⋯O4^i^	0.99	2.55	3.3351 (10)	137
C14—H14*A*⋯O5^ii^	0.99	2.66	3.1700 (12)	112
C8—H8*A*⋯O1^iii^	0.99	2.66	3.3775 (13)	130

Symmetry codes: (i) 
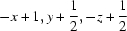
; (ii) 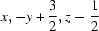; (iii) 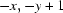, 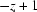.

**Fig. 1.**
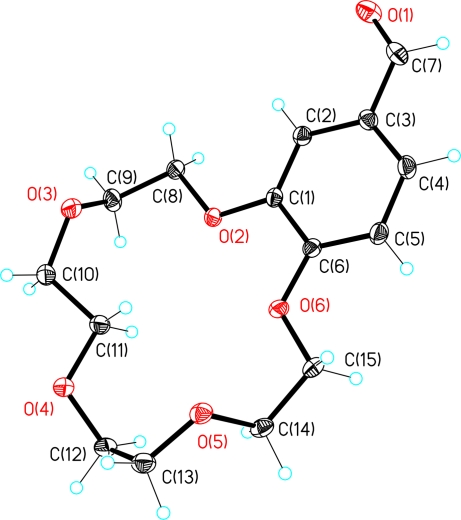
Molecular structure of the title compound with ellipsoids drawn at the 50% probability level.

**Fig. 2.**
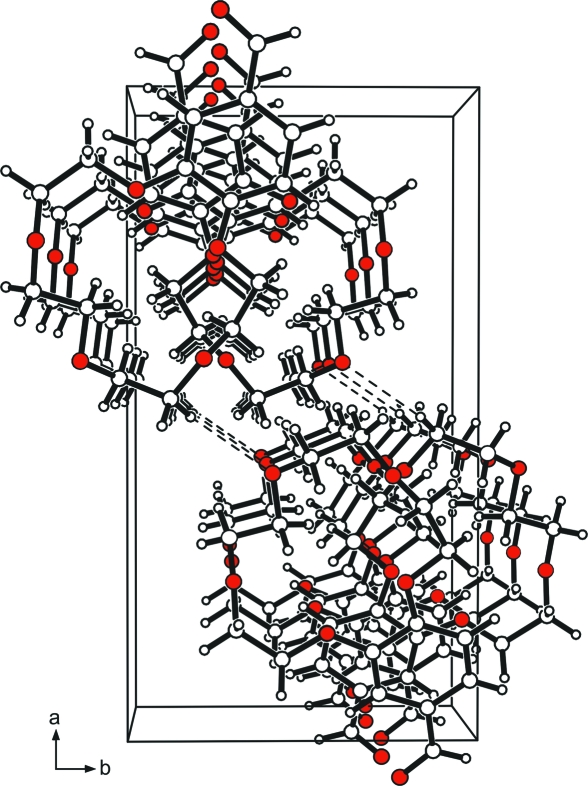
Packing diagram, viewed down the c axis.

## supplementary crystallographic information

### Comment

The title compound is a derivative of benzo-15-crown-5 (Pedersen, 1967). It was prepared as part of our studies concerning fluorogenic receptor molecules with possible analytical applications. The O-C-C-O torsion angles within the polyether ring are (±)gauche [69.34° (10), -71.10°(8), -65.61°(11)) and anti (168.42°(9)]) resulting in a twisted crown ether conformation. In the title molecule, the dihedral angle between the aromatic ring plane and the mean plane of ether oxygen atoms is 20.67 (5)°. Worth to note, the torsion angle C3—C4—C7—O1 is 179.75 (10)°, indicating only a very small twist of the formyl group relative to the aromatic ring. Thus, in agreement with a previous report (Rogers, Huggins *et al.*, 1992; Rogers, Henry & Rollins, 1992), the substituent on the benzene ring has negligible influence on the conformation of the benzo-15-crown-5 (Hanson, 1978). Owing to the absence of strong hydrogen bond donors, the crystal packing is stabilized by weak C—H···O hydrogen bonds, involving the O atoms of the crown ether and C==O group as acceptors, and the methylene C-H groups as donors (Table 1). In addition, π—π interaction has also been detected, resulting in a stacking of the molecules along the crystallographic *c* axis with a distance of 4.211 (2) Å between the centroids of two neighboring aromatic rings (Fig.2).

### Experimental

The title compound, 4'-formylbenzo-15-crown-5, was synthesized from benzo-15-crown-5 (Pedersen, 1967) which was reacted with *N*-methylformanilide and phoshoryl chloride (Hyde *et al.*, 1978). Colourless needles of the title compound suitable for X-ray diffraction analysis were obtained by slow cooling and evaporation of a solution of *n*-heptane. Fast cooling of the solution resulted in the formation of an orthorhombic polymorph of the title compound.

### Refinement

The H atoms were positioned geometrically and allowed to ride on their parent atoms, with C—H = 0.95 -0.99 Å, and *U*_iso_=1.2–1.5 *U*_eq_ (C).

### Crystal data

**Table:**

C_15_H_20_O_6_	*F*_000_ = 632
*M**_r_* = 296.31	*D*_x_ = 1.395 Mg m^−^^3^
Monoclinic, *P*2_1_/*c*	Mo *K*α radiation λ = 0.71073 Å
Hall symbol: -P 2ybc	Cell parameters from 9008 reflections
*a* = 18.0091 (8) Å	θ = 2.4–33.3º
*b* = 9.6678 (4) Å	µ = 0.11 mm^−^^1^
*c* = 8.1028 (3) Å	*T* = 90 (2) K
β = 91.262 (2)º	Plate, colourless
*V* = 1410.42 (10) Å^3^	0.60 × 0.39 × 0.05 mm
*Z* = 4	

### Data collection

**Table:**

Bruker Kappa APEXII CCD diffractometer	4523 independent reflections
Radiation source: fine-focus sealed tube	3716 reflections with *I* > 2σ(*I*)
Monochromator: graphite	*R*_int_ = 0.027
*T* = 90(2) K	θ_max_ = 31.1º
φ and ω scans	θ_min_ = 3.1º
Absorption correction: multi-scan(SADABS; Sheldrick, 2004)	*h* = −26→23
*T*_min_ = 0.857, *T*_max_ = 0.995	*k* = −14→12
18190 measured reflections	*l* = −11→11

### Refinement

**Table:**

Refinement on *F*^2^	Secondary atom site location: difference Fourier map
Least-squares matrix: full	Hydrogen site location: inferred from neighbouring sites
*R*[*F*^2^ > 2σ(*F*^2^)] = 0.037	H-atom parameters constrained
*wR*(*F*^2^) = 0.116	*w* = 1/[σ^2^(*F*_o_^2^) + (0.0681*P*)^2^ + 0.3911*P*] where *P* = (*F*_o_^2^ + 2*F*_c_^2^)/3
*S* = 1.00	(Δ/σ)_max_ < 0.001
4523 reflections	Δρ_max_ = 0.28 e Å^−^^3^
190 parameters	Δρ_min_ = −0.22 e Å^−^^3^
Primary atom site location: structure-invariant direct methods	Extinction correction: none

### Special details

**Table:**

Geometry. All e.s.d.'s (except the e.s.d. in the dihedral angle between two l.s. planes) are estimated using the full covariance matrix. The cell e.s.d.'s are taken into account individually in the estimation of e.s.d.'s in distances, angles and torsion angles; correlations between e.s.d.'s in cell parameters are only used when they are defined by crystal symmetry. An approximate (isotropic) treatment of cell e.s.d.'s is used for estimating e.s.d.'s involving l.s. planes.
Refinement. Refinement of *F*^2^ against ALL reflections. The weighted *R*-factor *wR* and goodness of fit *S* are based on *F*^2^, conventional *R*-factors *R* are based on *F*, with *F* set to zero for negative *F*^2^. The threshold expression of *F*^2^ > σ(*F*^2^) is used only for calculating *R*-factors(gt) *etc*. and is not relevant to the choice of reflections for refinement. *R*-factors based on *F*^2^ are statistically about twice as large as those based on *F*, and *R*- factors based on ALL data will be even larger.

### Fractional atomic coordinates and isotropic or equivalent isotropic displacement parameters (Å^2^)

**Table:**

	*x*	*y*	*z*	*U*_iso_*/*U*_eq_	
O1	−0.03932 (4)	0.76211 (8)	0.74777 (10)	0.02453 (17)	
O2	0.18803 (4)	0.54926 (7)	0.45384 (9)	0.01606 (14)	
O3	0.25972 (4)	0.29979 (8)	0.55829 (10)	0.02217 (17)	
O4	0.42078 (4)	0.40116 (8)	0.32578 (9)	0.01872 (15)	
O5	0.41719 (4)	0.72035 (7)	0.34545 (8)	0.01730 (15)	
O6	0.26420 (4)	0.75557 (7)	0.36194 (9)	0.01609 (14)	
C1	0.15926 (5)	0.67447 (9)	0.49388 (11)	0.01361 (17)	
C2	0.09428 (5)	0.69566 (10)	0.57629 (11)	0.01540 (17)	
H2	0.0650	0.6190	0.6084	0.018*	
C3	0.07132 (5)	0.83094 (10)	0.61289 (11)	0.01618 (18)	
C4	0.11385 (6)	0.94299 (10)	0.56708 (12)	0.01868 (19)	
H4	0.0984	1.0340	0.5940	0.022*	
C5	0.17961 (6)	0.92303 (10)	0.48118 (12)	0.01756 (18)	
H5	0.2085	1.0002	0.4487	0.021*	
C6	0.20225 (5)	0.78964 (9)	0.44389 (11)	0.01396 (17)	
C7	0.00239 (5)	0.85268 (11)	0.70205 (12)	0.02018 (19)	
H7	−0.0109	0.9455	0.7262	0.024*	
C8	0.14823 (5)	0.43015 (9)	0.50768 (13)	0.01689 (18)	
H8A	0.0984	0.4269	0.4539	0.020*	
H8B	0.1422	0.4329	0.6288	0.020*	
C9	0.19286 (5)	0.30593 (10)	0.46010 (14)	0.0203 (2)	
H9A	0.1633	0.2209	0.4769	0.024*	
H9B	0.2052	0.3116	0.3418	0.024*	
C10	0.32608 (5)	0.28738 (10)	0.46689 (14)	0.0204 (2)	
H10A	0.3193	0.2140	0.3827	0.025*	
H10B	0.3672	0.2589	0.5425	0.025*	
C11	0.34702 (5)	0.42146 (10)	0.38202 (12)	0.01686 (18)	
H11A	0.3126	0.4408	0.2879	0.020*	
H11B	0.3453	0.4999	0.4604	0.020*	
C12	0.44456 (5)	0.49820 (11)	0.20613 (12)	0.01883 (19)	
H12A	0.4024	0.5185	0.1295	0.023*	
H12B	0.4844	0.4554	0.1410	0.023*	
C13	0.47328 (5)	0.63335 (11)	0.27812 (12)	0.01947 (19)	
H13A	0.5106	0.6122	0.3662	0.023*	
H13B	0.4988	0.6851	0.1905	0.023*	
C14	0.37553 (5)	0.79418 (10)	0.22314 (11)	0.01733 (18)	
H14A	0.3570	0.7297	0.1367	0.021*	
H14B	0.4074	0.8642	0.1705	0.021*	
C15	0.31127 (5)	0.86389 (10)	0.30465 (11)	0.01632 (18)	
H15A	0.3291	0.9217	0.3982	0.020*	
H15B	0.2840	0.9236	0.2247	0.020*	

### Atomic displacement parameters (Å^2^)

**Table:**

	*U*^11^	*U*^22^	*U*^33^	*U*^12^	*U*^13^	*U*^23^
O1	0.0197 (3)	0.0272 (4)	0.0269 (4)	−0.0004 (3)	0.0049 (3)	−0.0043 (3)
O2	0.0180 (3)	0.0094 (3)	0.0211 (3)	−0.0007 (2)	0.0051 (2)	0.0003 (2)
O3	0.0173 (3)	0.0243 (4)	0.0250 (4)	0.0025 (3)	0.0044 (3)	0.0089 (3)
O4	0.0157 (3)	0.0198 (3)	0.0208 (3)	0.0011 (2)	0.0037 (2)	0.0046 (3)
O5	0.0187 (3)	0.0205 (3)	0.0127 (3)	−0.0001 (3)	0.0001 (2)	0.0022 (2)
O6	0.0177 (3)	0.0122 (3)	0.0186 (3)	−0.0023 (2)	0.0052 (2)	0.0007 (2)
C1	0.0164 (4)	0.0108 (4)	0.0137 (4)	0.0004 (3)	−0.0004 (3)	0.0002 (3)
C2	0.0164 (4)	0.0140 (4)	0.0158 (4)	−0.0003 (3)	0.0005 (3)	0.0001 (3)
C3	0.0175 (4)	0.0163 (4)	0.0148 (4)	0.0023 (3)	−0.0001 (3)	−0.0014 (3)
C4	0.0233 (4)	0.0129 (4)	0.0199 (4)	0.0028 (3)	0.0012 (3)	−0.0017 (3)
C5	0.0222 (4)	0.0119 (4)	0.0186 (4)	−0.0003 (3)	0.0011 (3)	0.0007 (3)
C6	0.0159 (4)	0.0133 (4)	0.0127 (4)	−0.0004 (3)	0.0002 (3)	0.0006 (3)
C7	0.0195 (4)	0.0209 (5)	0.0201 (4)	0.0039 (3)	0.0009 (3)	−0.0053 (4)
C8	0.0161 (4)	0.0112 (4)	0.0235 (5)	−0.0019 (3)	0.0035 (3)	0.0015 (3)
C9	0.0171 (4)	0.0124 (4)	0.0314 (5)	−0.0006 (3)	0.0021 (4)	−0.0001 (4)
C10	0.0169 (4)	0.0171 (4)	0.0274 (5)	0.0023 (3)	0.0044 (3)	0.0054 (4)
C11	0.0162 (4)	0.0154 (4)	0.0191 (4)	−0.0003 (3)	0.0033 (3)	−0.0001 (3)
C12	0.0193 (4)	0.0218 (5)	0.0156 (4)	0.0005 (3)	0.0043 (3)	0.0017 (3)
C13	0.0156 (4)	0.0230 (5)	0.0199 (4)	−0.0019 (3)	0.0023 (3)	0.0025 (4)
C14	0.0185 (4)	0.0208 (4)	0.0127 (4)	−0.0019 (3)	0.0009 (3)	0.0043 (3)
C15	0.0194 (4)	0.0141 (4)	0.0156 (4)	−0.0040 (3)	0.0008 (3)	0.0027 (3)

### Geometric parameters (Å, °)

**Table:**

O1—C7	1.2168 (13)	C7—H7	0.9500
O2—C1	1.3589 (11)	C8—C9	1.5001 (13)
O2—C8	1.4296 (11)	C8—H8A	0.9900
O3—C10	1.4249 (12)	C8—H8B	0.9900
O3—C9	1.4297 (13)	C9—H9A	0.9900
O4—C12	1.4219 (12)	C9—H9B	0.9900
O4—C11	1.4275 (11)	C10—C11	1.5189 (13)
O5—C14	1.4219 (12)	C10—H10A	0.9900
O5—C13	1.4318 (12)	C10—H10B	0.9900
O6—C6	1.3516 (11)	C11—H11A	0.9900
O6—C15	1.4311 (11)	C11—H11B	0.9900
C1—C2	1.3755 (12)	C12—C13	1.5171 (15)
C1—C6	1.4202 (12)	C12—H12A	0.9900
C2—C3	1.4052 (13)	C12—H12B	0.9900
C2—H2	0.9500	C13—H13A	0.9900
C3—C4	1.3822 (13)	C13—H13B	0.9900
C3—C7	1.4651 (13)	C14—C15	1.5044 (13)
C4—C5	1.4003 (13)	C14—H14A	0.9900
C4—H4	0.9500	C14—H14B	0.9900
C5—C6	1.3877 (13)	C15—H15A	0.9900
C5—H5	0.9500	C15—H15B	0.9900
			
C1—O2—C8	116.64 (7)	H9A—C9—H9B	108.2
C10—O3—C9	114.84 (8)	O3—C10—C11	112.51 (8)
C12—O4—C11	115.04 (7)	O3—C10—H10A	109.1
C14—O5—C13	113.27 (7)	C11—C10—H10A	109.1
C6—O6—C15	118.83 (7)	O3—C10—H10B	109.1
O2—C1—C2	125.56 (8)	C11—C10—H10B	109.1
O2—C1—C6	114.66 (8)	H10A—C10—H10B	107.8
C2—C1—C6	119.78 (8)	O4—C11—C10	105.62 (7)
C1—C2—C3	119.90 (9)	O4—C11—H11A	110.6
C1—C2—H2	120.0	C10—C11—H11A	110.6
C3—C2—H2	120.0	O4—C11—H11B	110.6
C4—C3—C2	120.36 (9)	C10—C11—H11B	110.6
C4—C3—C7	120.03 (9)	H11A—C11—H11B	108.7
C2—C3—C7	119.61 (9)	O4—C12—C13	114.29 (8)
C3—C4—C5	120.35 (9)	O4—C12—H12A	108.7
C3—C4—H4	119.8	C13—C12—H12A	108.7
C5—C4—H4	119.8	O4—C12—H12B	108.7
C6—C5—C4	119.47 (9)	C13—C12—H12B	108.7
C6—C5—H5	120.3	H12A—C12—H12B	107.6
C4—C5—H5	120.3	O5—C13—C12	114.52 (8)
O6—C6—C5	125.67 (8)	O5—C13—H13A	108.6
O6—C6—C1	114.21 (8)	C12—C13—H13A	108.6
C5—C6—C1	120.12 (8)	O5—C13—H13B	108.6
O1—C7—C3	125.61 (10)	C12—C13—H13B	108.6
O1—C7—H7	117.2	H13A—C13—H13B	107.6
C3—C7—H7	117.2	O5—C14—C15	108.54 (7)
O2—C8—C9	106.94 (7)	O5—C14—H14A	110.0
O2—C8—H8A	110.3	C15—C14—H14A	110.0
C9—C8—H8A	110.3	O5—C14—H14B	110.0
O2—C8—H8B	110.3	C15—C14—H14B	110.0
C9—C8—H8B	110.3	H14A—C14—H14B	108.4
H8A—C8—H8B	108.6	O6—C15—C14	106.35 (7)
O3—C9—C8	109.85 (8)	O6—C15—H15A	110.5
O3—C9—H9A	109.7	C14—C15—H15A	110.5
C8—C9—H9A	109.7	O6—C15—H15B	110.5
O3—C9—H9B	109.7	C14—C15—H15B	110.5
C8—C9—H9B	109.7	H15A—C15—H15B	108.7
			
C8—O2—C1—C2	−2.33 (13)	C2—C1—C6—C5	1.40 (14)
C8—O2—C1—C6	177.50 (8)	C4—C3—C7—O1	179.75 (10)
O2—C1—C2—C3	178.83 (9)	C2—C3—C7—O1	−1.06 (16)
C6—C1—C2—C3	−0.99 (14)	C1—O2—C8—C9	−176.14 (8)
C1—C2—C3—C4	−0.25 (14)	C10—O3—C9—C8	−127.42 (9)
C1—C2—C3—C7	−179.43 (9)	O2—C8—C9—O3	69.34 (10)
C2—C3—C4—C5	1.12 (15)	C9—O3—C10—C11	73.81 (11)
C7—C3—C4—C5	−179.71 (9)	C12—O4—C11—C10	164.16 (8)
C3—C4—C5—C6	−0.71 (15)	O3—C10—C11—O4	168.42 (8)
C15—O6—C6—C5	−1.01 (14)	C11—O4—C12—C13	82.80 (10)
C15—O6—C6—C1	179.48 (8)	C14—O5—C13—C12	−78.28 (10)
C4—C5—C6—O6	179.97 (9)	O4—C12—C13—O5	−71.10 (11)
C4—C5—C6—C1	−0.54 (14)	C13—O5—C14—C15	171.27 (8)
O2—C1—C6—O6	1.10 (12)	C6—O6—C15—C14	178.69 (8)
C2—C1—C6—O6	−179.06 (8)	O5—C14—C15—O6	−65.61 (9)
O2—C1—C6—C5	−178.45 (8)		

### Hydrogen-bond geometry (Å, °)

**Table:**

*D*—H···*A*	*D*—H	H···*A*	*D*···*A*	*D*—H···*A*
C13—H13B···O4^i^	0.99	2.55	3.3351 (10)	137
C14—H14A···O5^ii^	0.99	2.66	3.1700 (12)	112
C8—H8A···O1^iii^	0.99	2.66	3.3775 (13)	130

Symmetry codes: (i) −*x*+1, *y*+1/2, −*z*+1/2; (ii) *x*, −*y*+3/2, *z*−1/2; (iii) −*x*, −*y*+1, −*z*+1.
